# Dynamics of land use land cover change and its effect on urban heat island in Halaba Kulito Town

**DOI:** 10.1016/j.heliyon.2025.e41689

**Published:** 2025-01-05

**Authors:** Begonet Dale, Mihret Dananto, Bisrat Kifle

**Affiliations:** a Faculty of Urban Development & Engineering, Ethiopian Civil Service University (ECSU), Addis Ababa, Ethiopia; b Schools of Biosystem & Environmental Engineering, Hawassa University (HU), Hawassa, Ethiopia

**Keywords:** LULC, Supervised, TerrSet, Classification accuracy, Halaba Kulito Town, Urban heat island

## Abstract

Halaba Kulito Town in Ethiopia has experienced significant urbanization over the past three decades, leading to the conversion of natural land into built-up environments, causing environmental deterioration and impacting the local climate. This study set out to look at the dynamics of land use and cover change and how they have affected Halaba Kulito Town's Urban Heat Island during the previous thirty years. The analysis used the Landsat imagery (30 m × 30 m) of 1991, 2001, 2011, and 2021. The supervised classification approach was used to classify the images. The evaluation of classification accuracy was checked using a confusion error matrix. For the years 1991, 2001, 2011, and 2021, the LULC change classification accuracy evaluation showed results of 70.81 %, 81.3 %, 81 %, 91 %, and 0.71, 0.81, 0.81, and 0.89, respectively, for overall classification accuracy and kappa coefficient. In 2001, 2011, and 2021, the built-up increased by 23.6 %, 31.6 %, and 43.13 %, respectively. Over the first two decades, from 1991 to 2001 and 2001 to 2011, the quantity of land under cultivation increased by 15.7 % and 14.3 %, respectively, but dropped by 59.18 % from 2011 to 2021. Between 1991 and 2001, range land dropped by 33.3 %; nevertheless, in the next two decades, it expanded by 3.3 % and 83.2 %. The amount of bare land declined by 67.1 % between 2001 and 2011, although it increased by 38.8 % between 1991 and 2001 and 3.36 % between 2011 and 2021. In addition, wetland increased by 47.1 % and 191.95 % between 2011 and 2021 and between 1991 and 2001, respectively, while declining by 62 % between 2001 and 2011. Compared to the baseline 1991-year aerial coverage, built-up, range land, and wetland expanded by 132.9 %, 26.2 %, and 63.3 % over the previous thirty years, whereas bare land and cultivated land dropped by 46 % and 52.8 %, respectively. The mean urban heat island (UHI) for 1991, 2001, 2011, and 2021 incorporating seasonal influence was 0.003, 0.002, 0.005, and 0.00, respectively. This is because the intensity of the UHI is correlated with changes in land use and land cover (LULC). In contrast, the lowest UHI dropped by 35.3 % in 2021 compared to the value in 1991, while the maximum UHI increased by 18.6 % from its 1991 level. In comparison to 1991, the mean intensity of UHI increased by 106.21 % and 120.4 %, respectively, in 2011 and 2021, whereas it declined by 20.67 % in 2001. The findings of this study offer decision makers with scientifically proven knowledge about land use and its implications for the urban heat island effect. It will serve as a guideline for developing and implementing integrated land use management to produce climate resilient cities and create livable urban environments for citizens.

## 1 Introduction

Natural ecosystems offer numerous goods and services but are often neglected in planning, leading to harm. Land use and land cover change (LULCC) disrupts ecosystem functions like regulation, habitat, production, and aesthetics in many countries [1]. The International Geosphere-Biosphere Programme (IGBP), the International Human Dimensions Programs on Global Environmental Change (IHDP), the National Research Council of the National Academies series Board on Earth Sciences and Resources (BERSR), and the Division on Earth and Life Studies (DELS) have all highlighted the significant impact of land use and land cover change on the environment [2]. Human activities, such as agricultural land expansion, forest product demand, settlement expansion, and urbanization, have led to a significant increase in land use and land cover disturbances over the past six decades. This has resulted in the alteration of 30 %–50 % of environmental ecosystems, causing ecological degradation and environmental pollution [1,3,4]. According to Refs. [1,3,5] explanations, about 30 %–50 % of natural ecosystems have been changed over the past 50 years more than ever through land use and land cover change.

Both natural and man-made variables, such as agriculture, forest fire, overgrazing, urbanization, and climate change, have an impact on changes in land use. However, human-induced causative elements have been more widely recognized. Human induced elements include agricultural land expansion, timber exploitation, and infrastructural development and settlements. Arable land and settlement areas are replacing large portions of naturally vegetated landscape in Ethiopia. It makes them susceptible to drought, floods, environmental deterioration, and soil erosion [6,7]. The urban area is in challenge of global warming, flooding, and the urban heat island (UHI) effect on a local scale.

In this study, the focus was on the LULC change and effect on the UHI over the past three decades. Hence, the conceptualization was concentrated on land use, land cover change, and the UHI parameters. The LULC changes typical of the expansion of impervious surfaces such as buildings, asphalt, parking lots, paved and concrete roads, and other surfaces have been increasing land surface temperature (LST) at local, regional, and global levels [8]. LST is one of the environmental elements that is exposed to the effects of land use and land cover change. It is explained as follows by Ref. [9]: “The LST measures the surface temperature at the canopy level for vegetation, at the soil surface for barren land, and at the surface layers for urban land cover types' '. The increase in LST has a consequence of rising Urban Heat Island (UHI) compared to the surrounding rural area. Urban heat island (UHI) refers to the phenomenon that urban areas tend to have higher atmospheric or surface temperatures than their surroundings [10,11]. The UHI has a significant negative effect on economic development (for example, net primary production), environment (biodiversity, water, and air quality), climate, and generally on human health and well-being (increasing morbidity, mortality, and risk of violence) [8,11]. Hence, understanding land use, land cover change, and its effect on the local urban heat island is very important for sustainable environmental management and the implementation of climate change mitigation and resilience strategies.

According to different scholars in Ethiopia, the LULC change was significant even though the rate and extent of the change varied with spatial and temporal variations. For example, according to Ref. [12], in the Blue Nile Basin, the cultivated land was expanded at Kuhar Michael while decreasing at Lenche Dema watersheds. Shrubland and grassland decreased at Kuhar Michael but increased Lenche Dema watershed over the years 1973–2005. In Ref. [13] study, agriculture and shrub land were expanded by 60 % and 12 % between 1989 and 2016 in the area around Adama Town. According to Ref. [14], cultivated land and settlement were expanded by 67.38 % and 53.2 %, while decreasing of forest, shrub, and grassland by 66.35 % and 18.36 % over the years 1972–2017, respectively, at Halaba - Bilate sub watershed. The [15] study in Western Ethiopia has revealed that forest land decreased from a proportion of 69 % in 1978 to 13 %, 8.5 %, and 6.5 % in 1991, 2010, and 2016, respectively, while agricultural land increased by 13 % between 2013 and 2016. Other scholars, such as [[16], [17], [18], [19], [20]], and [21], have indicated that the built-up area has expanded significantly over the past three decades in Addis Ababa, Hawassa, Debre Markos, Mekele, and Bahir Dar cities in Ethiopia.

Ethiopia has experienced unpredictable land use and cover changes over the past three decades, particularly in the Dire-Dawa, Assosa, Jigijiga, and Gambella Towns. This expansion has led to increased temperature and urban heat, affecting the towns frequently. On the other hand, managers and professionals face challenges in managing uncontrolled conversion of land use and make the towns resilient to climate change.

Even though LULC change detection is not a new research area in Ethiopia, the effect of the change on the UHI effect has not been researched yet. In fact, some scholars, for example [22], tried to analyze the impact of landscape variation on LST in Addis Ababa; [23], tried to analyze LST in Bahir Dar city without relating the change of LULC and its impact; and [8], tried to see the LST also with regard to green space in Addis Ababa, Hawassa, Bahir Dar, and Adama cities. Among the examined studies, no one attempted to relate land use and land cover change to the UHI impact. The LULC change and its influence on the local climate have not been studied in medium-sized towns like Halaba Kulito in Ethiopia. As a result, studying LULC changes and their impact on local development is critical for developing appropriate land use management strategies, implementing climate change adaptation and mitigation activities to protect urban ecology and community health, and fostering sustainable economic development.

Halaba kulito is one of the capital cities of Ethiopia's Central Regional State, situated in the central rift valley basin. It is a new town that has been witnessing an alarming pace of population growth and horizontal expansion in built-up areas. However, the lack of previous research on land use and land cover dynamics in Halaba kulito, as well as the link to urban heat islands, posed a challenge to decision-makers, urban planners, and environmentalists in achieving sustainable development goals in the local context. This study was aimed at analyzing the status of land use and land cover change in the town and its effect on the urban heat island. The finding fills decision-making gaps through providing scientifically proven information on the issues. Therefore, the land use land cover status over the past three-decade period (1991–2021) was detected using 1991, 2001, 2011, and 2021 remotely sensed Landsat imagery. To come up with the intended result, the following objectives were targeted:

• To examine the land use and land cover change in Halaba Kulito Town over the past three decades (1991–2021) using the 1991, 2001, 2011, and 2021 Landsat imagery.
• To examine the dynamics of the changes in land use over the period using the TerrSet Geospatial Monitoring and Modeling system.
• To analyze the effect of the LULC change on UHI over the Town

## 2 Material and methods

### 2.1 Description of study

**Fig. 2.1:**
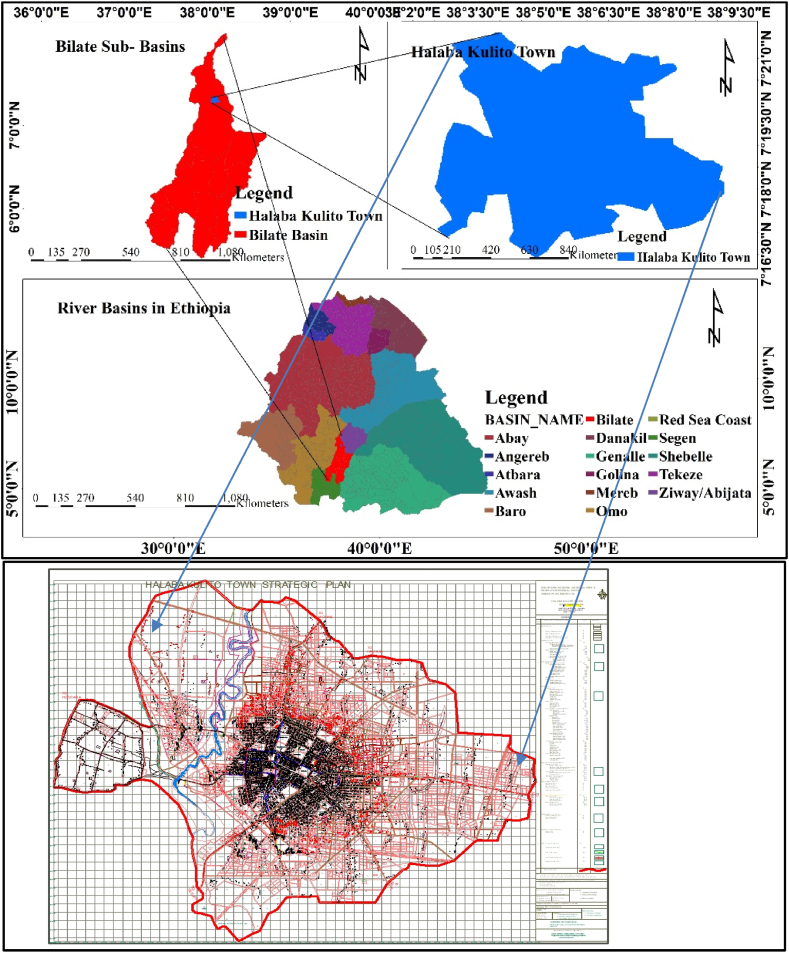
Location of the study area. [Source: SNNPR^1^ Urban Development Bureau, 2021].

The study area is found in the Central Rift Valley of Ethiopia, in the northern part of Bilate Sub-Basin. It is located between 7° 17′ 19" and 7° 19′ 25" N and 38° 4′ 10" and 38° 6′ 17" E. The overall area of the research area comprises 6946 ha (96.5 km^2^). Its altitude ranged from 1554 to 2149m a.s.l. Fig. 2.1 indicates the location of the Town and its structural plan. The Town is located south of Addis Ababa, the capital city of Ethiopia, at 243 km across Butajira Town.

Agro-ecologically, Halaba Kulito Town is characterized as a subtropical zone, and it experiences both a hot and cold climate and a bimodal rainfall pattern, which is the long rainy season “kiremt” (June to September) and the short rainy season “belg” season (February to May). The dry season, “Bega,” ranges from October to February. Its annual mean rainfall ranges from 544 mm to 1271 mm, whereas the temperature is 12 °C to 25 °C [24,25]. The area is dominantly characterized by three main seasons: “Bega” (October–January), which is the dry season; “Belg” (February–May), a short rain season; and “Kiremt” (June–September), a long rain season similar to the national seasonal circumstances [26].

**Fig. 2.2:**
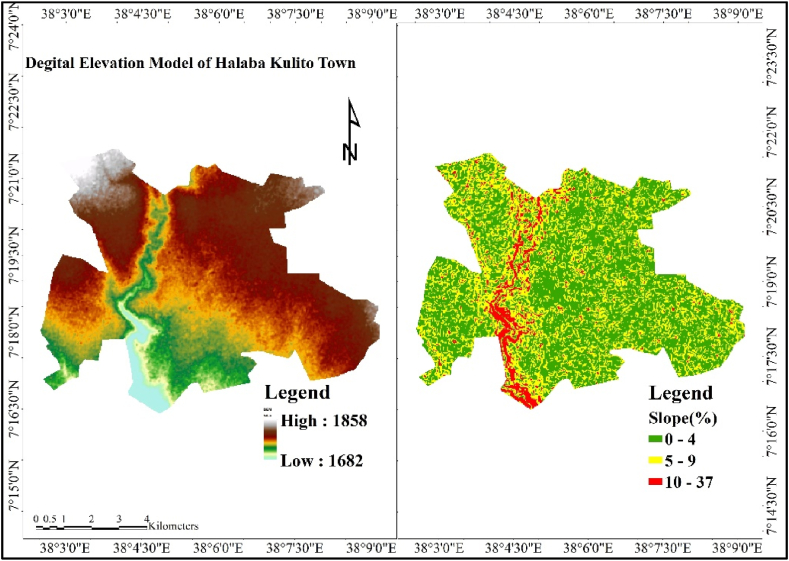
DEM and slope of the study area. [Source: Extracted from USGS].

The pixel size of 30 by 30 SRTM DEM was used to calculate the slope of the study area, and the slope ranges between 0 and 37 % (https://earthexplorer.usgs.gov/). The majority of the area under study has a flat, gentle, and moderate slope of gradient. According to Iaaich et al. (2016), the UNEP/FAO approach to slope classes explains that the area under the gradient (%) 12 is gentle and moderate with an elevation of 1682–1859 m a.s.l. (Fig. 2.2).

### 2.2 Descriptions and data sources

The goal of this research was to detect LULC change in the Town and analyze its dynamics over the decades. The past three-decade state of land use was analyzed using a sample of 1991, 2001, 2011, and 202 Landsat images with 30 × 30 resolutions. The state of the LULC change was described and quantified; therefore, the descriptive and explanatory type of research design was applied to execute the required analysis. For this type of research, according to Ref. [27] explanation, the suitable approach is the application of quantitative methods. Therefore, a quantitative research approach was utilized in this study to detect the change and its dynamics and analyze the effects on the urban heat island.

The data that was used for the study were the strategic map of the Town, the land use map, the digital elevation model (DEM), the Landsat landSat5, and the OLI8 ground truth coordinates. Both primary and secondary data were applicable for the study. The primary data was collected through field observation and surveying, whereas secondary data was obtained from satellite imagery and documents. The strategic plan of the town was collected from the SNNPR Urban Development Bureau, Landsat, and Dem acquired from the United States Geological Survey (USGS) source; the land use map was obtained from the Ministry of Water and Energy of Ethiopia; and the ground truth data was collected through field surveying.

### 2.3 Data analysis

**Fig. 2.3:**
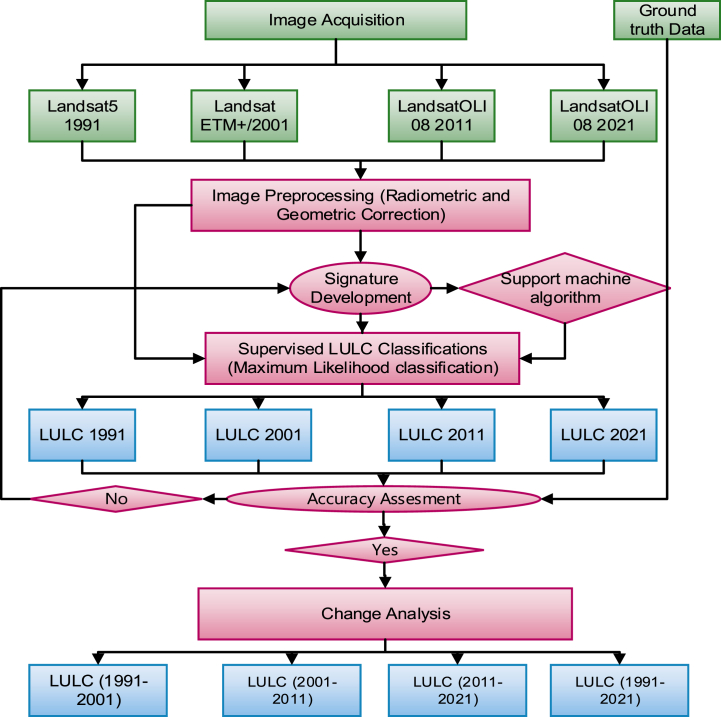
Land use land change detection framework.

The LULC change detection and its dynamics were analyzed using the TerrSet geospatial monitoring and modeling software. The process has begun with the selection of the appropriate input data for the detection of historic conditions of LULC. The input data were Landsat 5 of 1991, 2001, and operational land imager (OLI08) of 2011 and 2021 imagery. After acquiring the 1991, 2001, 2011, and 2021 imagery of the study area from USGS, image preprocessing such as image mosaicking and composite was conducted. Then classification of the image was done under the supervised classification method by developing training samples. The classification accuracy was checked using an accuracy assessment matrix using the ground truth and exported from Google Earth data. The data analysis was conducted by following the steps in Fig. 2.3 below.

Land covers nomenclatures must be first identify and characterize prospective land cover classes in order to simplify sample collecting and classification. The land cover classes applied in this paper are adopted from Anderson Classification System (ACS) (Table 2.1).

**Table 2.1:** Land use land cover classes adopted from Anderson Classification System (ACS).

Class Name	Description
Urban or built-up	Residential, Commercial and services, Industrial, Transportation, communications, and utilities, Industrial and commercial complexes, Mixed urban or built-up land, and Other urban or built-up land
Agricultural land	Cropland and pasture, Orchards, groves, vineyards, nurseries, and ornamental horticultural areas, and Confined feeding operations
Rangeland	Herbaceous rangeland, Shrub and brush rangeland and Mixed rangeland
Forestland	Deciduous forest land, Evergreen forestland, Mixed forestland
Water	Streams and canals, Lakes, Reservoirs, Bays and estuaries
Wetland	Forested wetland, No forested wetland
Barren land	Dry salt flats, Beaches, Sandy areas other than beaches, Bare exposed rock, Strip mines, quarries, and gravel pits, Transitional areas, and Mixed barren land

Image acquisition, preparation and processing, land use classification, accuracy assessment, change detection, and analysis were the main steps that were passed throughout the processes.

#### 2.3.1 Image acquisition and preprocessing

**Fig. 2.4:**
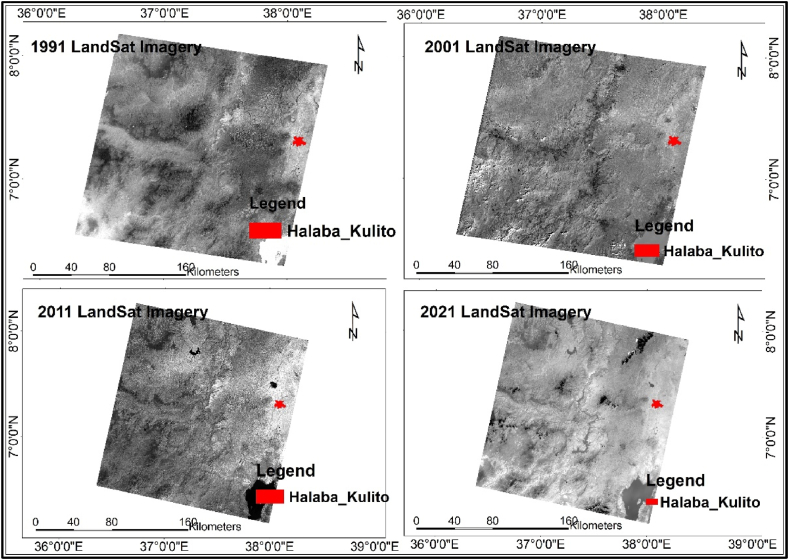
Landsat imagery. [Source, USGS website].

Remote sensing data for detecting land cover change relies on spatial, temporal, spectral, and radiometric resolution qualities. Common options include IKONOS, Landsat TM and ETM+, SPOT, and high-resolution radiometers and MODIS sensors [29]. Researchers for example [17,30,31], have utilized Landsat images to detect land use land cover change in different parts of Ethiopia. Therefore, based on the previous studies, cost and its availability Landsat imagery was chosen for this study as indicated in Table 2.2 and Fig. 2.3. Landsat imagery with 30 m resolution for the 1991, 2001, 2011, and 2021, as well as a 30 m resolution digital elevation model was collected from https://earthexplorer.usgs.gov/. The image acquisition years in Ethiopia were influenced by the 1991 deposition of the communist military regime by the Ethiopian People Republic Development Front-led government, which led to a shift in land use policy, as noted by Refs. [32,33]. Based on this information the past three-decade land use of the Town was analyzed starting from 1991 to 2021 by using remote sense imagery of 1991, 2001, 2011 and 2021 (Table 2.2) and Fig. 2.4 below.

**Table 2.2:** Landsat imager description.

Satellite Image	Path/Row	Acquisition year	Spatial resolution	Band number
Landsat05_L1TP	169/055	1991-12-28	30 m	7
Landsat05_L1TP	169/055	2001-12-15	30 m	7
Landsat08_L2SP	169/055	2011-12 08	30m	7
Landsat08_L2SP	169/055	2021-01-05	30m	7
DEM

According to Ref. [34] the acquisition period of the imageries would have significant seasonal variation and affect the land use classification quality. To avoid such the seasonal variation, the dry season was selected since the dry season in Ethiopia is a cloud-free time [33].

Before analysis the images must be pre-processed because they are subject to errors. Geometric correction and radiometric correction are the two primary pre-processing steps.

##### 2.3.1.1 Geometric correction

Geometric corrections are preprocessing procedures that are often necessary before analyzing images because there might be a discrepancy in an image between its real coordinates and the ideal coordinates that, in theory, would be projected using the best possible sensor and environment [34]. Therefore, to avoid the distortions from a distorted image a step which was identified was applied.

##### 2.3.1.2 Radiometric correction

The image quality may be affected by distortion clouds. Such distortion and error could be reduced by stacking image layers, filtering composites, and mosaicking. According to Ref. [35] each sensor has its own calibration parameters the DN values; the same DN values in two images taken by two different sensors may actually represent two different radiance values. Therefore, the radiance calibration will be conducted using the following equations:

(2.1) $$L=\left(\frac{L_{\max}-L_{\min}}{255}\right)DN+L_{\min}$$

where, $L=the\;radiance\;\frac{w}{m^{2}\left(sr\right)}\left(watt\;per\;square\;meter\;per\;steradian\right)\;\frac{mw}{{cm}^{2}sr\left(mm\right)}$

$$L_{\mathrm{Max}}=highest\;DN\;value\;L_{\min}=Lowest\;DN\;value$$

##### 2.3.1.3 False color composition

The images were composited in order to select the best composition using the TerrSet Geospatial Monitoring and Modeling system. As a result, the false color composite imagines generated from Landsat 8 bands 5-4-3 (Red-Green-Blue), Landsat TM bands 4-3-2 (Red-Green-Blue), and ETM+ 4-3-2 (Red-Green-Blue).

#### 2.3.2 Image classifications methods

Satellite images are used for land use classification, with two methods: supervised and unsupervised. Supervised classification requires users to assign training data for predetermined land use and cover classes, with the effectiveness of categorization based on user training and data availability. Unsupervised classification doesn't require user selection of training data sets [27,29]. Ethiopian scholars, including [17], and [14], prefer supervised techniques over traditional classification methods due to their higher accuracy. Hence, in this study the supervised classification method was applied under maximum likelihood probability.

Various methods like maximum likelihood, random forest, support vector machines, and convolutional neural networks are used for land use and land cover classification. According to Ref. [36] maximum likelihood is a widely used parametric classifier in LULC classification, measuring the Gaussian distribution of spectral classes with a covariance matrix. Random Forest, based on multi-classification decision trees, is particularly effective for accurately mapping land cover in complex and varied terrains [37]. Support Vector Machine (SVM) is also a widely used machine learning algorithm for remote sensing image classification, achieving higher accuracy in multitemporal satellite image classification. It uses classification and regression to find the best hyperplane for a given sample but it is object-based classification [38].

The comparison between the maximum likelihood and support vector machine algorism the both method of classification enjoys higher and appropriate accuracy in classification. However maximum likelihood method is commonly used in land use land cover classification in Ethiopia. For example [18,20], used this method to classify land use land cover change in Bahir-Dar whereas [31] applied in Halaba- Billate sub catchment. Due to this reason the maximum likelihood method of land use change prediction was conducted in this typical study.

#### 2.3.3 Land use classification accuracy assessment

According to Refs. [28,39] developing an error matrix is a common method to assess classification accuracy. Hence, the error matrix was developed by collecting ground truth points and using Google Earth data to associate and verify the spectral classes of each LULC map in this research using ArcGIS 10.7 software. The method was used by different scholars; for example [33], used the error matrix to test the overall, user, and producer accuracy of the classification. The overall accuracy is calculated by dividing the total number of correctly classified pixels by the total number of reference pixels (eq. (2.2)).

(2.2) $$OA=\frac{TS}{TCS}*100$$

The user's accuracy is calculated by dividing the correctly classified number of pixels by the number claimed to be in that map (eq. (2.3)).

(2.3) $$UA=\frac{{CC}_{R}}{TR\;}*100$$

The producer accuracy is calculated by dividing correctly identified numbers in reference plots of a given class by total number in that class (eq. (2.4)).

(2.4) $$PA=\frac{{CC}_{C}}{TC}*100$$

The Kappa Coefficient is denoted by $\hat{k}$ and calculated by dividing the difference between observed accuracy and chance agreement by one minus chance agreement (eq. (2.5)) and its rating statistics was tabulated in Table 2.3.

(2.5) $$\hat{k}=\frac{\left(TD*TCS\right)-\sum \left(TC*TR\right)}{{TS}^{2}-\sum \left(TC*TR\right)}$$

where,

OA=overallaccuracy

TS=Totalnumberofcorrectlyclassifiedpixel(diagonal)

TCS=Totalnumberofreferencepixel

UA=users’accuracy

$${CC}_{R}=Correctly\;Classified\;Pixel\;in\;each\;row$$

TR=totalpixelintherow

$$PA=Producer^{\prime }s\;Accuracy$$

$${CC}_{C}=Correctly\;Classified\;Pixel\;in\;each\;column$$

TC=totalpixelinthecolumn.

**Table 2.3:** Rating statistics of kappa coefficient.

No.	Kappa statistics	Strength of Agreement
1	<0	Poor
2	0.01–0.40	Slight
3	0.41–0.60	Moderate
4	0.61–0.80	Substantial
5	0.81–1.00	Almost Perfect

#### 2.3.4 The change analysis

The change analysis was performed using the Land Change Modeler (LCM) panel in TerrSet geospatial monitoring and modeling software. The panel allows importing the earlier and later land use images in LCM session parameters. Then spatial character harmonizing and dimension adjustment were done based on either an earlier or later land cover image spatial character. The adjustment enables the user to run the model and detect the changes (gained and lost, net change by category, and contributors to net change). Finally, the change dynamics and rate of change over 1991–2021 years were calculated using the empirical relationship (eq. (2.6) and eq. (2.7))

(2.6) $$\%\;of\;Change=\frac{{LULC}_{L}-{LULC}_{E}}{{LULC}_{E}}$$

(2.7) $$Rate\;of\;Change\;\left(\frac{ha}{year}\right)=\frac{{LULC}_{L}-{LULC}_{E}}{T}$$

where, ${LULC}_{E}=is\;the\;area\;of\;LULC\;\left(ha\right)\;of\;an\;earlier\;land\;cover\;image$

$${LULC}_{L}=is\;the\;area\;of\;LULC\;\left(ha\right)\;of\;a\;later\;land\;cover\;image$$

$$T=the\;time\;interval\;between\;{LULC}_{E}\;and\;{LULC}_{L}\;in\;years$$

The change dynamics was calculated between the land use classes and over the period (1991–2001, 2001–2011, & 2011–2021). The significance of the change between the land uses and over the time period was analyzed using ANOVA at α=5% significance level.

#### 2.3.5 Land use land cover change drivers

Land use land cover change influenced by different driving factors include natural and human activities (Gashaw et al., 2017). Natural drivers’ effect for example, climate change effect is felt after extended periods of time whereas human driver is effects are immediate. The population growth over the past three decade was analyzed using the empirical equation at equation eq. (2.8) below

(2.8) $$r=\frac{1}{n}\ln\frac{P_{t2}}{P_{t1}}*100$$

where, r = growth rate in percent, $P_{t2}=$ the population at time 2, $P_{t1}=$ the population at time 1, and n is the number of years between time 1 and time 2.

The study examined the correlation between population growth and land use and land cover in Halaba Kulato Town using population data in 1994 and 2007 census and 2022 projected reports by central statistics authority of Ethiopia.

#### 2.3.6 Urban heat island effect (UHI) analysis

Urban heat island effect analysis over the past three decades due to land use and land cover change was executed by following the formulae from 8 to 14. Various scholars, for example [[41], [42], [43]], and [44], used the following equations to calculate the land surface temperature and urban heat island. Therefore, in this method, all six steps described below were used for the determination of the urban heat island in the study area.

Step 1 In this step the top of atmosphere (TOA) radiance of the imagery was calculated Using the radiance rescaling factor, Thermal Infra-Red Digital Numbers can be converted to TOA spectral radiance (eq. (2.9)).

(2.9) Lλ=ML∗Qcal+AL

where: Lλ = TOA spectral radiance (Watts/(m2 ∗ sr ∗ μm)), ML = Radiance multiplicative Band (No.), AL = Radiance Add Band (No.), Qcal = Quantized and calibrated standard product pixel values (DN).

Step 2 The top of atmosphere (TOA) brightness temperature was calculated by converting spectral radiance data to top of atmosphere brightness temperature using the thermal constant Values in metadata file (eq. (2.10)).

(2.10) BT=K2/ln(k1/Lλ+1)−272.15

where: BT = Top of atmosphere brightness temperature (°C), Lλ = TOA spectral radiance (Watts/(m2 ∗ sr ∗ μm)), K1 = K1 Constant Band (No.), K2 = K2 Constant Band (No.)

Step 3 Normalized Differential Vegetation Index (NDVI) which is a standardized vegetation index Calculated using Near Infra-red (Band 5) and Red (Band 4) bands (eq. (2.11)).

(2.11) NDVI=(NIR–RED)/(NIR+RED)

where: RED = DN values from the RED band, NIR = DN values from Near-Infrared band.

Step 4 Land Surface Emissivity (LSE): is the average emissivity of an element of the surface of the Earth and was calculated from NDVI values in (eq. (2.12)) and PV value in eq. (2.13) below.

(2.12) $$PV={\left[\frac{\left(NDVI\;–\;NDVI\;\min\right)}{\left(NDVI\;\max+NDVI\;\min\right)}\right]}^{2}$$

where: PV = Proportion of Vegetation, NDVI = DN values from NDVI Image, NDVI min = Minimum DN values from NDVI Image, NDVI max = Maximum DN values from NDVI Image

(2.13) E=0.004∗PV+0.986

where: E = Land Surface Emissivity, PV = Proportion of Vegetation.

Step 5 Land Surface Temperature (LST) is the radiate temperature calculated using Top of atmosphere brightness temperature, Wavelength of emitted radiance, Land Surface Emissivity (eq. (2.14))

(2.14) PLST=(BT/1)+W∗(BT/14380)∗ln(E)

where: BT = Top of atmosphere brightness temperature (°C), W = Wavelength of emitted radiance, E = Land Surface Emissivity.

Step 6 Urban Heat Island (UHI) Calculation was finally determined using the formula in equation (14) in ArcGIS 10.8 based on the empirical formula (2.15)

(2.15) $$UHI=\frac{\left(LST-{LST}_{m}\right)\;}{Sd}$$

where, UHI = Urban Heat Island, LST = Land Surface Temperature (°C), ${LST}_{m}$ = the mean land surface temperature of the study area, and SD = standard deviation of the temperature

## 3 Result and discussion

### 3.1 LULC change analysis (1991–2021)

#### 3.1.1 Land use land cover change accuracy assessment

The classification accuracy assessment was 70.81 %, 81.3 %, 81 %, 91 %, and 0.71, 0.81, 0.81, and 0.89, respectively, for overall classification accuracy and kappa coefficient for the years 1991, 2001, 2011, and 2021 (Table 3.1).

**Table 3.1:** Classification accuracy assessment confusion matrix/.

1991							
*LULC*	*BU*	*CL*	*RL*	*BL*	*WL*	*TR*	*UA (%)* = $UA=\frac{{CC}_{R}}{TR}*100$
*BU*	*18*	*2*	*0*	*0*	*0*	*20*	*90*
*CL*	*0*	*20*	*3*	*2*	*0*	*25*	*80*
*RL*	*1*	*3*	*17*	*2*	*0*	*23*	*73.9*
*BL*	*2*	*2*	*0*	*15*	*0*	*19*	*78.9*
*WL*	*4*	*0*	*0*	*2*	*14*	*20*	*70*
*TC*	*25*	*27*	*20*	*21*	*14*	*107*	
*PA (%)* = $UA=\frac{{CC}_{C}}{TC}*100$	*90.9*	*74.1*	*85.0*	*71.4*	*100.0*		*OA* = $\frac{TS}{TCS}=\frac{87}{107}$ = *70.81* *%*

*2001*							
*LULC*	*BU*	*CL*	*RL*	*BL*	*WL*	*TR*	*UA (%)* = $UA=\frac{{CC}_{R}}{TR}*100$

*BU*	*20*	*2*	*0*	*0*	*0*	*22*	*90.9*
*CL*	*2*	*20*	*3*	*0*	*0*	*25*	*80.0*
*RL*	*0*	*5*	*18*	*0*	*0*	*23*	*78.3*
*BL*	*0*	*2*	*0*	*15*	*0*	*17*	*88.2*
*WL*	*0*	*0*	*4*	*2*	*14*	*20*	*70.0*
*TC*	*22*	*29*	*25*	*17*	*14*	*107*	
*PA (%)* = $UA=\frac{{CC}_{C}}{TC}*100$	*90.9*	*69.0*	*72.0*	*88.2*	*100.0*		*OA* = $\frac{TS}{TCS}=\frac{91}{107}$ = *81.3* *%*

*2011*							
*LULC*	*BU*	*CL*	*RL*	*BL*	*WL*	*TR*	*UA (%)* = $UA=\frac{{CC}_{R}}{TR}*100$

*BU*	*21*	*2*	*0*	*0*	*0*	*23*	*91.3*
*CL*	*0*	*27*	*4*	*1*	*0*	*32*	*84.4*
*RL*	*1*	*4*	*15*	*0*	*0*	*20*	*75.0*
*BL*	*3*	*2*	*0*	*10*	*0*	*15*	*66.7*
*WL*	*2*	*1*	*0*	*0*	*10*	*13*	*76.9*
*TC*	*27*	*36*	*19*	*11*	*10*	*103*	
*PA (%)* = $UA=\frac{{CC}_{C}}{TC}*100$	*78*	*75*	*79*	*91*	*100*		*OA* = $\frac{TS}{TCS}=\frac{83}{103}$ = *81* *%*

*2021*							
*LULC*	*BU*	*CL*	*RL*	*BL*	*WL*	*TR*	*UA (%)* = $UA=\frac{{CC}_{R}}{TR}*100$

*BU*	*24*	*0*	*0*	*1*	*0*	*25*	*96.0*
*CL*		*21*	*2*	*3*		*25*	*84.0*
*RL*	*0*	*1*	*17*	*2*	*1*	*20*	*85.0*
*BL*	*1*	*0*	*0*	*14*	*0*	*15*	*93.3*
*WL*	*0*	*2*		*0*	*15*	*15*	*100.0*
*TC*	*25*	*24*	*19*	*20*	*16*	*100*	
*PA (%)* = $UA=\frac{{CC}_{C}}{TC}*100$	*96.0*	*87.5*	*89.5*	*70.0*	*93.8*		*OA* = $\frac{TS}{TCS}=\frac{91}{100}$ = *91* *%*

Where, LULC= Land Use Land Cover, BU=Built-up, CL= Cultivated Land, RL = Rangeland, BL= Bare land, WL= Wetland, TR = Total Row, TC = Total Column.

#### 3.1.2 Land use land cover change detection

Over the past three decades, the built-up has increased by 23.6 %, 31.6 %, and 43.13 % in 2001, 2011, and 2021, respectively. Cultivated land was expanded by 15.7 % and 14.3 % over the first two consecutive decades between 1991 and 2001 and 2001 and 2011 but decreased by 59.18 % from 2011 to 2021. Range land decreased by 33.3 % from 1991 to 2001 but increased by 3.3 % and 83.2 % between 2001 to 2011 and 2011 to 2021. Bare land decreased by 67.1 % between 2001 and 2011, but increased by 38.8 % between 1991 and 2001 and by 3.36 % in the year between 2011 and 2021. Between 1991–2001 and 2011–2021, the wetland area expanded by 47.1 % and 191.95 %, respectively, whereas it declined by 62 % between 2001 and 2011.

**Fig. 3.1:**
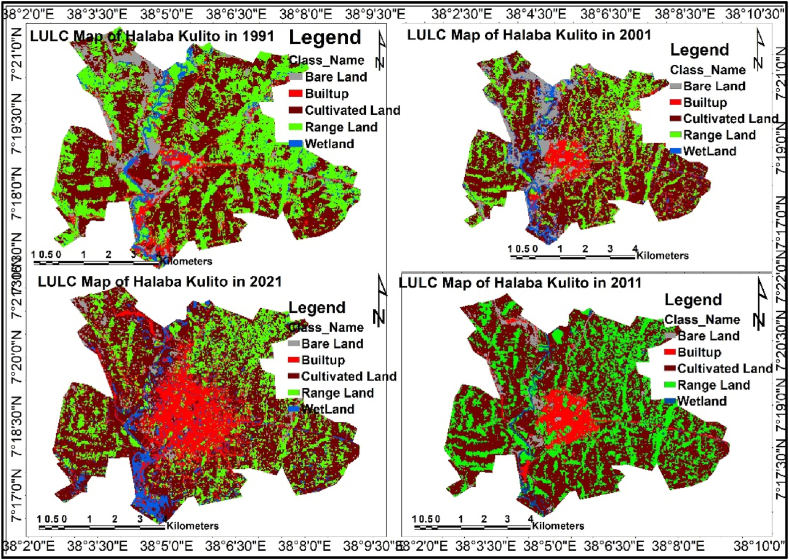
Halaba Kulito LULC map (1991, 2001, 2011, 2021).

The overall change result from 1991 to 2021 has shown that built-up, range land, and wetland were increased by 132.9 %, 26.2 %, and 63.3 %, respectively, whereas cultivated land and barren land were decreased by 46 % and 52.8 % compared with the baseline 1991-year aerial coverage. The built-up area increased more than 100 % within the past thirty years, primarily in the cost of cultivated and barren land (Table 3.2 and Fig. 3.1).

**Table 3.2:** Land use land cover of Halaba Kulito town.

Class Name	Area (Hectare)				Change in (%)			
	1991	2001	2011	2021	1991–2001	2001–2011	2011–2021	1991–2021
*BU*	*517.5*	*640*	*842*	*1205*	*23.7*	*31.6*	*43.13*	*132.9*
*CL*	*2887.2*	*3341*	*3820*	*1560*	*15.7*	*14.3*	*−59.18*	*−46.0*
*RL*	*2737.8*	*1825*	*1886*	*3455*	*−33.3*	*3.3*	*83.20*	*26.2*
*BL*	*504.4*	*700*	*230*	*238*	*38.8*	*−67.1*	*3.36*	*−52.8*
*WL*	*299.1*	*440*	*167*	*488*	*47.1*	*−62.0*	*191.95*	*63.3*

The study has shown similar result with regard to built-up area result [14] study in Halaba Bilate sub catchment, but unaligned with regard to cultivated area result since their study result says that the cultivated land was expanded between 1972 and 2017. However, it was consisted with [45] who said that built-up areas between 1991 and 2018 have been steadily expanding while accelerating decline in cultivated land in Bahir Dar Town.

The reason behind the expansion of built-up areas is the architecture of the legal framework increasing of population. The constitution of Federal Democratic Republic of Ethiopia, which was approved in 1995, and land acquisition Proclamation No456/2005, Article. 5 sub-Article 1a permitting free and unlimited access to land; have contributed to the increase of expansion of settlement and the decline of bare land. Even the documents appreciated that “every citizen from 18 years of age who wants to make a living from agriculture should be given free access to land” [46]. On the other hand [47,48], explained that the government of Ethiopia has not put in place an integrated national land use policy; hence, the situation has aggravated the change from cultivated and bare land to built-up. The [49] also predicted urban expansion will be expected by the loss of crop land by 1.8–2.4 % in 2030, which will be significant in new emerging Towns and cities in Asia and Africa.

Halaba Kulito Town is among the newly emerging Towns and leveled as large Towns according to the revised structural plan manual of Ministry of urban development and construction of Ethiopia in 2012. The share of built-up area was increasing from 7.5 % of the total area in 1991 to 9.2 % in 2001, 12.12 % and 17.36 % in 2021, respectively. The expansion of built-up area is a threat for natural disasters (flooding) since it hinders the infiltration capacity. According to Ref. [50], built-up area expansion is related with an increase in population. Hence this aggravates environmental pollutants in the future and environmental hazards will be strong threats to the residents.

#### 3.1.3 Significances test of overall land use land cover change

The study utilized the ANOVA method to test the significance of changes in land use class and time intervals, as per [51] method, which tests the level of differences between means. The F and P values were used to determine the significance of the changes (Table 3.3).

**Table 3.3:** Significance test of land use land cover change.

ANOVA						
*Source of Variation*	*SS*	*df*	*MS*	*F*	*P-value*	*F crit*
*Land use classes*	*0.15*	*3*	*0.05*	*0.00*	*1*	*3.49*
*Time interval*	*0.00*	*4*	*5841612.54*	*13.67*	*0.0002*	*3.25*
*Error*	*5124647*	*12*	*427053.94*			
*Total*	*0.00*	*19*				

Rows = years (1991, 2001, 2011 & 2021), Columns = land Uses (built-up, cultivated land, rangeland, Bare land & wetland).

According to the table of 3.4, the *F crit (3.25)* of the columns is less than the calculated *F (13.67)* and the *P-value (0.002)* is less than the alpha value at 95 % of confidence interval. This illustrates the net change either an expansion or declining between the land use classes varied significantly at the Town over the past three decades (1991–2021).

### 3.2 Dynamics of LULC change

#### 3.2.1 Gain and loss of LULC

**Fig. 3.2:**
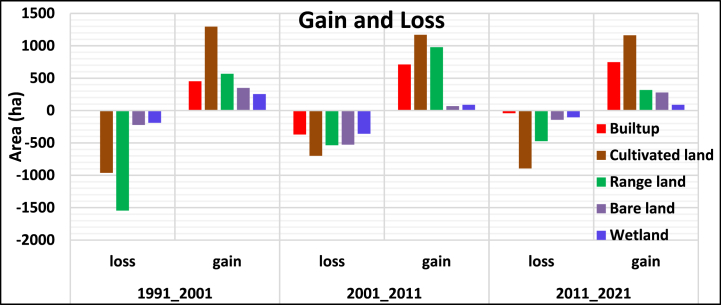
Gain and losses of LULC

According to the summary of the results in Table 3.4 and Fig. 3.2, the LULC change result has shown that built-up, cultivated land, rangeland, bare land, and wetland gained 451, 1295.7, 567.9, 349.9, and 254.3 ha in 2001, while cultivated land, rangeland, bare land, and wetland lost 961.7, 1546, 221.4, and 189.7 ha. In the year between 2001 and 2011, built-up, cultivated land, range, bare land, and wetlands gained 710.6, 1169, 978, 53, and 72 ha, respectively, while losing 370.26, 700, 536, 527, and 358 ha. Between 2011 and 2021, the amount of built-up, cultivated, bare, and wet land gained 746, 1161, 317, 273, and 85 ha while losing 42, 894, 472, 144, and 105 ha. Between 1991 and 2001, wetland gained the least amount, whereas cultivated land experienced the largest amount of gain. On the other hand, rangeland lost a lot, whereas there was no loss from built-up.

**Table 3.4:** Gain & loss of land use over the period (ha).

Land use Classes	1991_2001		2001_2011		2011_2021	
	Loss	Gain	Loss	Gain	Loss	Gain
*Built-up*	*0*	*451*	*370.26*	*710.6*	*42*	*746*
*Cultivated land*	*961.7*	*1295.7*	*700*	*1169*	*894*	*1161*
*Range land*	*1546.0*	*567.9*	*536*	*978*	*472*	*317*
*Bare land*	*221.4*	*349.9*	*527*	*53*	*144*	*273*
*Wetland*	*189.7*	*254.3*	*358*	*72*	*105*	*85*

#### 3.2.2 Land use gain and loss significance

Based on the statistical values of the losses and gains of the land use between the specified time intervals was tested and the result was summarized in Table 3.5 below.

**Table 3.5:** ANOVA of gain and loss.

additional land gain	ANOVA					
*Source of Variation*	*SS*	*df*	*MS*	*F*	*P-value*	*F crit*
*Land use classes*	*2132436.6*	*4*	*533109.14*	*13.02*	*0.001*	*3.84*
*Time intervals*	*19770.358*	*2*	*9885.18*	*0.24*	*0.79*	*4.46*
*Error*	*327590.56*	*8*	*40948.82*			
*Total*	*2479797.5*	*14*				
*Loss of land*						
*Land use Classes*	*1486096*	*4*	*371524.1*	*3.74*	*0.053*	*3.84*
*Time intervals*	*164741.7*	*2*	*82370.87*	*0.83*	*0.47*	*4.46*
*Error*	*795479.8*	*8*	*99434.98*			
*Total*	*2446318*	*14*				

Columns = years (1991, 2001, 2011 & 2021), Rows = land Uses (built-up, cultivated land, rangeland, Bare land & wetland).

According to Table 3.5, the gain value of land use classes *F crit (3.84)* is less than the calculated *F (13.02)* and the *P-value (0.001)* is less than the α=0.05 whereas the gained land with in time interval has F crit (4.46) is greater than the calculated *F (0.24)* and the *P-value (0.79)* is greater than the α=0.05. With regard to the losses of land between land uses class, the *F crit (3.84)* is greater than the calculated *F (3.74)* and the *P-value (0.053)* is greater than α=0.05 whereas the land use loss within time interval, F *crit (4.46)* is greater than the calculated *F (0.84)* and the *P-value (0.47)* is greater than α = 0.05.

The amount of land between land use classes varied significantly and it determined land use types whereas the gain variation was not significant due to time intervals over the past three decades (1991–2021) however, the loss of land between land use classes and within time interval was not varied significantly. Therefore, one can concluded as the variation of gain between the land use classes was determined by variation of land use types but not due to time interval variation but the loss variation was not significant due to both land use type and time interval. This necessitates the land use type-based land management strategies development to minimize uncontrolled change.

This result aligns with the study report of [52] which said that there was significant variation of land use change between the years 1990–2019 in Nashe watershed in Blue Nile basin.

#### 3.2.3 Contribution to the net change

##### 3.2.3.1 In built-up

**Fig. 3.3:**
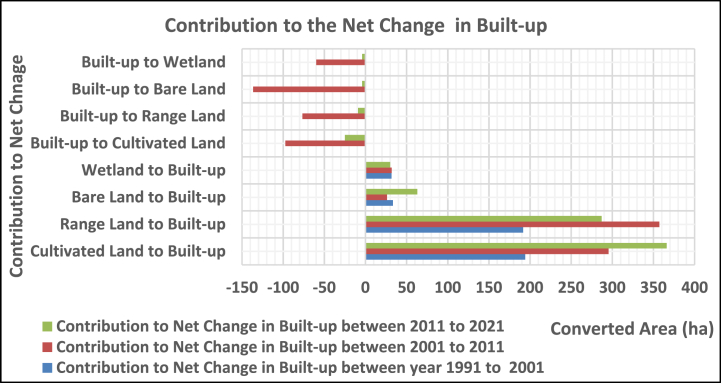
Contribution to net change in built-up.

The built-up area contributed 194.21, 191.64, 33.41, and 31.75 ha from cultivated rangeland, bare land, and wetland, respectively, between 1991 and 2001, whereas between 2001 and 2011, it gained 295, 357, 26, and 32 ha, respectively, while losing 97, 77, 137, and 60 ha to cultivation, rangeland, bare land, and wetland. In the year between 2011 and 2021, the built-up area contributed 366, 287, 63, and 30 ha from cultivated land, rangeland, bare land, and wetland, while losing 25, 9, 4, and 4 ha to cultivation, range, bare land, and wetland, respectively (Fig. 3.3).

According to Fig. 3.3, cultivate land and rangeland were the major contributors of additional land to built-up. This result aligns with the study by Ref. [33], who said that the built-up area was mainly contributed from agriculture, shrubs, and forest land.

##### 3.2.3.2 In cultivated land

**Fig. 3.4:**
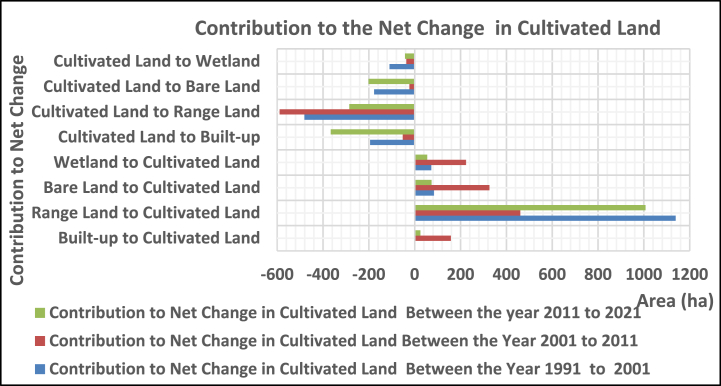
Contribution to net change in cultivated land.

Cultivation land was contributed with 1138.8ha, 84.4 ha, and 72.6 ha from range land, bare land, and wetland, while 194.2 ha, 481.0ha, 76.7 ha, and 109.8ha were built up from range land, bare land, and wetland between the years 1991 and 2001, respectively. Between 2001 and 2011, it contributed 158, 461, 326, and 224 ha of built-up land, rangeland, bare land, and wetland, while losing 52, 589, 23, and 36 ha to land use, respectively. It gained 25, 1007, 74, and 55 ha from built-up, rangeland, bare land, and wetland between the years 2011 and 2021, respectively, while losing 366 ha, 285, 201 ha, and 42 ha to land uses (Fig. 3.4).

##### 3.2.3.3 In rangeland

**Fig. 3.5:**
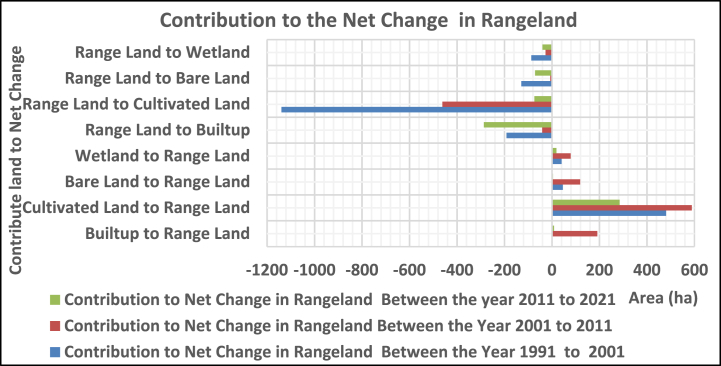
Contribution to net change in rangeland.

The range land was contributed with 481, 46.1, and 40.9 ha from cultivated, bare land, and wetlands, whereas it lost 191.6, 1138.8, 128.7, and 86.9 ha to built-up, cultivated, bare land, and wetlands, respectively, between 1991 and 2001. In the year between 2001 and 2011, it gained 191, 589, 119, and 79 ha of built-up, cultivated, bare land, and wetland, while giving 41, 461, 7, and 27 ha to land uses, respectively. Between 2011 and 2021, it contributed 9, 285, 4, and 19 ha of built-up, cultivated, bare land, and wetland, whereas it lost 287, 74, 71, and 40 ha to land uses, respectively (Fig. 3.5).

**Fig. 3.6:**
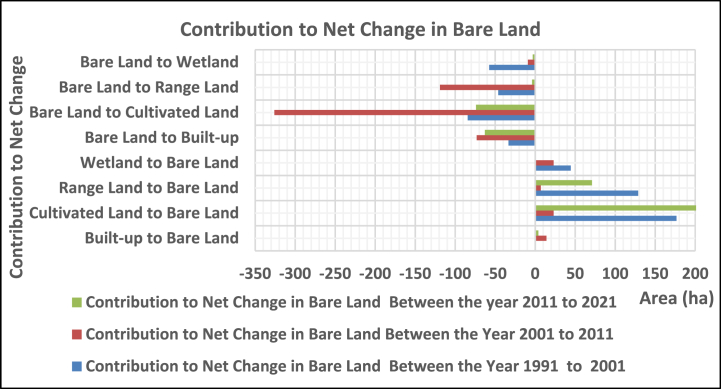
Contribution to net change in bare land.

In 1991–2021, the bare land was contributed with 176.7, 128.7, and 44.5 ha from cultivated, range, and wetland, while 33.4, 84.4, 46.1, and 57.6 ha were dominated to built-up, cultivated, range, and wetland, respectively. In 2001–2011, it gained 14, 23, 7, and 23 ha from built-up, cultivated, range, and wetland, while losing 73, 326, 119, and 9 ha to land uses, respectively. Between 2011 and 2021, the gain from built-up, cultivated land, range, and wetland was 4,201, 71, and 1 ha, while the loss was 63, 74, 4, and 3 ha, respectively (Fig. 3.6).

**Fig. 3.7:**
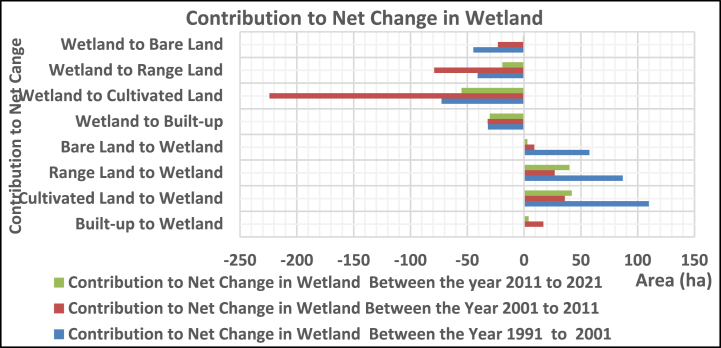
Contribution to net change for wetland.

The wetland was contributed from cultivated, rangeland, and bare land (109.8, 86.9, and 57.6 ha) while losing 31.8, 72.6, 40.9, and 44.5 ha to built-up, cultivated, rangeland, and bare land, respectively, from 1991 to 2001. Whereas gained 17, 36, 27, and 9ha from built-up, cultivated land, rangeland, and bare land while losing 32, 224, 79, and 23 ha to land use, respectively, between 2001 and 2011. In the year between 201 and 2021, it has gained 4, 42, 40, and 3 ha of built-up, cultivated, rangeland, and bare land, while losing 30, 55, 19, and 1 ha, respectively, to land uses (Fig. 3.7).

Hence, in the past three decades (1991–2021), built-up, range land, and wetland were increased by 132.9 %, 26.2 %, and 63.3 %, respectively, whereas cultivated land and bare land were decreased by 46 % and 52.8 %, respectively, compared with 1991-year area coverage. On average, over the past three decades, the gains by built-up, cultivated land, rangeland, bare land, and wetland were 635.9, 1208.6, 621.0, 231.3, and 144.1 ha, while losing 137.4, 851.9, 851.3, 297.5, and 217.6 ha on average. The result has shown that the impervious area or built-up area was expanded highly by the cost of cultivated land, range land, and wetland, respectively, compared to 1991. This has a significant effect on the natural environment and the local climate.

### 3.3 Land use land cover change drivers

The land use land cover change was accelerated due to different factors such as urbanization and lack of strong land use legislation [53]. Halaba Kulito officials acknowledge the horizontal expansion of land due to lack of urban land policy, but the increasing urbanization over the past three decades has also increased demand for large-scale land conversions (Koroso et al., 2021). Ethiopia's urbanization rate is low compared to sub-Saharan countries, but it experiences rapid growth, with a 4.2 % annual increase between 1994 and 2015 [54]. This specific study found that the town population increased by 6.77 % annually between 2007 and 2022 [55]. The results are consistent with those of [56], who argued that changes in land policy and population growth trends are important drivers of changes in land usage.

### 3.4 Land surface temperature (LST) and urban heat island (UHI)

**Fig. 3.8:**
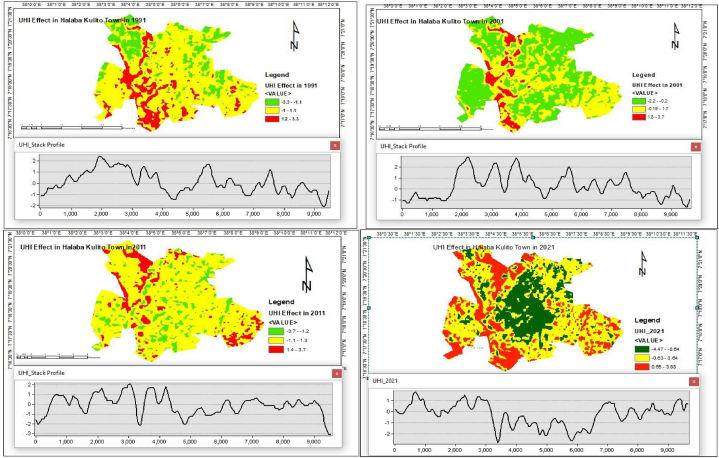
UHI effect in 1991, 2001, 2011 & 2021.

The results in Table 3.6 and Fig. 3.8 show that the estimated land surfaces mean temperature (LST) and urban heat island over the study area were extended from 1991 to 2021. The mean land surface temperature that was calculated from the Landsat imagery resulted in 29.50, 27.25, 31.52, and 66.37, whereas the mean urban heat island (UHI) was 0.003, 0.002, 0.005, and 0.00, respectively, for 1991, 2001, 2011, and 2021, including seasonal impact. The maximum UHI was increased by 18.6 % in 2021 from the level it was in 1991. On the other hand, the minimum UHI decreased by 35.3 % in 2021 compared to the value in 1991. The mean intensity of UHI increased by 106.21 % and 120.4 % in 2011 and 2021 compared to 1991, whereas it decreased by 20.67 % in 2001. The spatial variation of the UHI, as illustrated in Fig. 3.8, increases from the center to the edges of the Town.

**Table 3.6:** Land surface temperature and urban heat island over the town.

year	LST				UHI			
	TempMax	TempMin	Mean	Std.dev	Max	Min	mean	Std.dev
*1991*	*35.26*	*23.68*	*29.50*	*1.76*	*3.271*	*−3.305*	*0.003*	*0.999*
*2001*	*34.86*	*22.82*	*27.25*	*2.06*	*3.694*	*−2.153*	*0.002*	*0.998*
*2011*	*37.22*	*25.83*	*31.52*	*1.53*	*3.734*	*−3.715*	*0.005*	*1.001*
*2021*	*70.47*	*61.62*	*66.37*	*1.06*	*3.879*	*−4.473*	*0.006*	*1.001*

### 3.5 Land use land cover change vs. urban heat island

Between 1991 and 2001, there was a 20.7 % drop in the UHI due to an increase of built-up, cultivated, bare, and wet land by 23.7, 15.7 %, 38.8, and 47.1 %, respectively, and the decline of range land by 33.3 %. The expansion of alternative land uses had an impact on the UHI throughout the time, despite the built-up area growing. In comparison to bare land and wetland, which decreased by 67.1 % and 62 % between 2001 and 2011, the area of built-up, cultivated land, and range land increased by 31.6 %, 14.3 %, and 3.3 %, respectively. As a result, the change caused the UHI to rise by 159.9 %. In 2021, the area of built-up, rangeland, bare land, and wetland increased by 43.13 %, 82.2 %, 3.36 %, and 191.91 % in comparison to 2011, while the area of cultivated land decreased by 59.18 %. The UHI computation result indicates that at that time, it increased by 6.9 %. Thus, in comparison to the preceding two decadal changes from 1991 to 2021, the increase was smaller in percentage. Over the last three decades, the total area of built-up, rangeland, and wetland expanded by 132.9 %, 26.2 %, and 63.3 %, whereas the amount of cultivated land and bare land decreased by 46 % and 52.8 % at Halaba Kulito Town. As a result, during this time, the impact of these changes has caused a change in UHI of 120.4 %.

This study, conducted on a newly emerging town in Ethiopia, supports previous research by Refs. [8,22,23], which focused on the capital city and secondary cities. The findings also highlight the impact of land use and land cover change on land surface temperature and heat island effect in medium towns in Ethiopia, highlighting the importance of considering these factors in urban planning.

**Fig. 3.9:**
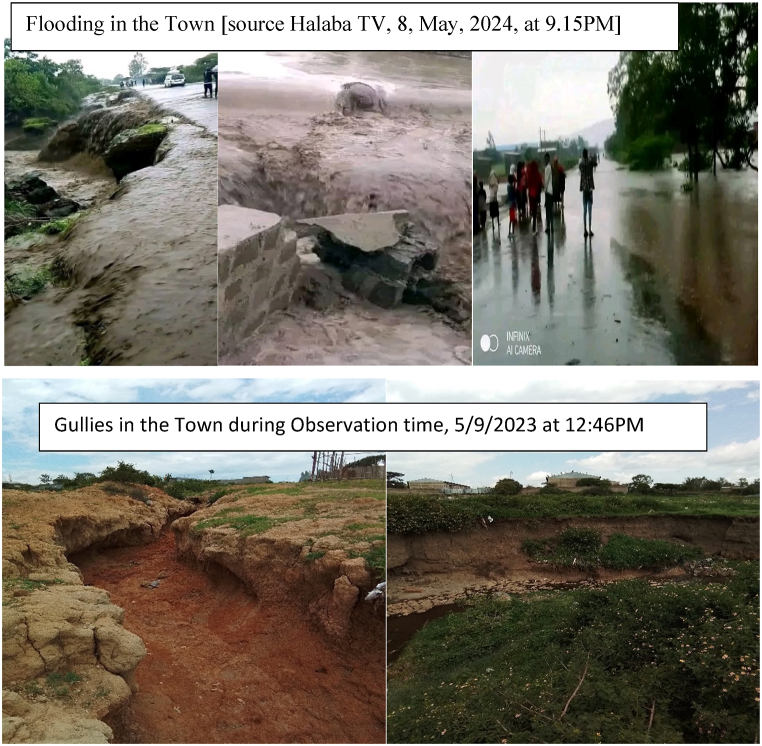
Some implication of land use land cover change.

The town experienced flooding, land degradation, and increased urban heat due to land use and land cover change, resulting in high runoff, soil erosion, and even flooding, resulting in seven deaths according to Crisis24 report (Fig. 3.9).

## 4 Conclusion

The land use and land cover have changed dynamically during the last thirty years. Since it has kept increasing over the course of three decades, the built-up area has been greatly extended at the expense of other land usage. Other land uses, such as rangeland and wetland, have shown an increasing trend compared to the 1991 area coverage, but cultivated land and bare land have decreased. Throughout the research period, there was a rise in both the land surface temperature and the urban heat island, suggesting that changes in land use and cover had an impact on the parameter increment. To maintain the environment as it is now, the Town must have an integrated land use plan and a natural environmental preservation policy.

## 5 Recommendation

Based on the findings of this study the following recommendation is forwarded for the local as well as national governments:

• Land usage Inventory and integrated planning must be implemented to protect uncontrolled conversion of land use;
• Promote strong advocacy on the integrated land use management and at community, individual level
• The town should also consider adopting law and policy enforcement tactics climate change adaptation and mitigation strategies has to be implemented at the local scale.

## CRediT authorship contribution statement

**Begonet Dale:** Writing – original draft, Methodology, Investigation, Funding acquisition, Formal analysis, Data curation, Conceptualization. **Mihret Dananto:** Validation, Supervision. **Bisrat Kifle:** Supervision.

## Data availability statement

Research data used for this study will be made available if requested by contacting the corresponding author.

## Declaration of competing interest

The authors declare that they have no known competing financial interests or personal relationships that could have appeared to influence the work reported in this paper.

## Footnotes

1 Southern Nations Nationalities and peoples Region.
